# A case of long QT syndrome: challenges on a bumpy road

**DOI:** 10.1002/ccr3.985

**Published:** 2017-05-04

**Authors:** Peter Magnusson, Per‐Erik Gustafsson

**Affiliations:** ^1^ Cardiology Research UnitDepartment of MedicineKarolinska InstitutetStockholmSE‐171 76Sweden; ^2^ Centre for Research and DevelopmentUppsala University/Region GävleborgGävleSE‐801 87Sweden

**Keywords:** Genetic, implantable cardioverter–defibrillator, long QT syndrome, pregnancy, premature ventricular complex, risk stratification, sudden cardiac death

## Abstract

Beta‐agonist treatment during pregnancy may unmask the diagnosis of long QT syndrome. The QT prolongation can result in functional AV block. A history of seizure and/or sudden death in a family member should raise suspicion of ventricular tachycardia. More than one mutation may coexist. Refusal of beta‐blocker therapy complicates risk stratification.

## Introduction

Long QT syndrome (LQTS) is linked to mutations in the ion channels, which can lead to disturbances in ventricular repolarization 1. This condition puts patients at risk for syncope due to polymorphic ventricular tachycardia, typically *torsades de pointes* (TdP) which may deteriorate into ventricular fibrillation and cause sudden cardiac death (SCD) 2. This report evaluates the case of a young woman who was diagnosed with LQTS during pregnancy and whose management involved a series of clinical challenges. The patient gave written informed consent to publish this account.

## Case History

**Figure 1:**
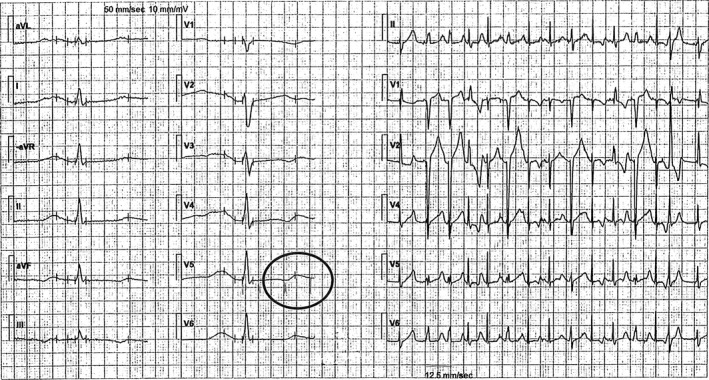
First ECG when referred for cardiologist consultation (on terbutaline). The QT is misinterpreted by the computerized interpretation, but manual measurement reveals QT far above 500 msec.

**Figure 2:**
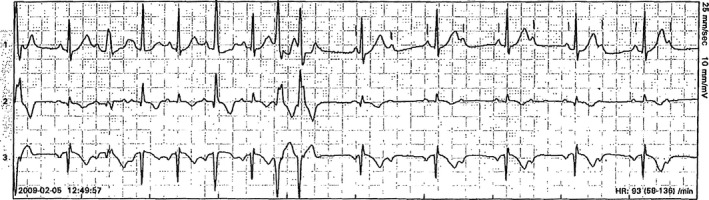
Ambulatory ECG when referred for cardiologist consultation (off terbutaline). Functional AV block, second degree, premature ventricular complex, and long QT.

In 2009, a pregnant woman taking phenytoin medication for epilepsy was referred to the cardiology clinic due to premature ventricular complexes (PVCs). She had a history of two miscarriages, which occurred in gestational weeks 8 and week 21, respectively, because of suspected cervical insufficiency. When she became pregnant again, a cerclage was inserted to avoid late miscarriage or preterm birth. The beta‐adrenergic agonist terbutaline was administrated intravenously to postpone preterm labor. She experienced palpitations, and her ECG was abnormal, revealing PVCs, atrioventricular (AV) 2:1 block and QT prolongation (520 msec) in precordial lead V_5_ during sinus rhythm at 90 beats per minute (Fig. 1) and rhythm strip while walking (Fig. 2). The PVCs had a monomorphic appearance and a left bundle branch block (LBBB) form in precordial lead V_1_, which indicates origin in the right chamber. A 24‐h ambulatory ECG confirmed frequent PVCs (10% of the time) and three runs of nonsustained ventricular tachycardia (three beats each), and five episodes of bigeminal PVCs. Long QT syndrome (LQTS) was suspected. Echocardiography, including right ventricular projections, excluded structural heart disease. An ergometer exercise test was scheduled, but the patient did not come to the appointment. The situation was complicated by the patient's anxiety, especially with regard to the healthcare environment.

Based on the established guidelines and expert opinions, she was at risk for SCD. The ECG finding of QT>500 msec plus T‐wave pathology suggestive for Long QT syndrome type 3 considered markers for an increased risk of SCD. In addition, pregnancy and the postpartum period are both known to be a vulnerable period for the patient's heart. Of particular concern to the patient was the fact that her own mother had died suddenly at 36 years of age. The mother died suddenly and was found on the kitchen floor after strenuous exercise at the gym. Our patient was referred to an experienced physician for the implantation of an implantable cardioverter–defibrillator (ICD), which she accepted. The implantation proceeded without complications and with limited fluoroscopy time. To protect her unborn child, ionizing radiation use was reduced. A Current™ DR RF 2207‐97 (St. Jude Medical, St. Paul, MN) was implanted subcutaneously in the left infraclavicular area and connected to a Durata™ 58 cm single‐coil defibrillation lead with active fixation (St Jude Medical). The Durata™ defibrillation lead was implanted in the lower septal portion of the right ventricle, and a Tendril™ 52 cm atrial pacing lead (St Jude Medical) was affixed in the right atrial appendage. Bradycardia pacing was programed to a base rate of 60 bpm and maximum tracking rate 130 bpm. Tachycardia detection was set to 240 bpm and 100 intervals before discharge of 42 J. The shock waveform was optimized based on the system's high‐voltage lead impedance of 70 Ohms and without T‐wave oversensing.

The patient became the index patient in the family for LQTS. Familial genetic screening took 4 months and was completed just a few weeks before the patient delivered her baby. The suspicion of LQTS was confirmed by the presence of a SCN5A mutation c.1231G>A in heterozygotic form.

At first, hypotension, systolic blood pressure of 90 mmHg, and concern for the fetus all prevented the initiation of beta‐blocker therapy which would have otherwise been ordered, but later on when these concerns were no longer at issue, the patient still refused a daily dose of metoprolol 50 mg.

She reported seven episodes of loss of consciousness between an age of 13 and 16 but no episodes since then. The patient had been diagnosed in childhood with epilepsy, but we questioned this diagnosis in light of her LQTS combined with the absence of any electroencephalography pathology and an atypical history of seizure. Nevertheless, we advised her to continue with her phenytoin 200 mg twice daily, which she did. Phenytoin affects sodium channels and we considered these effects to be of benefit to her arrhythmias, although this constitutes an off‐label use of phenytoin. The pregnancy proceeded, the cervical cerclage was removed, and she gave birth to a son. During the normal vaginal delivery, she was monitored with pulse oximetry and no significant arrhythmias were reported. Pediatricians evaluated the son with an ECG, and the baby was referred for genetic screening. It turned out that he has the same LQTS1 mutation as his mother. A cascade screening of other family members was performed, and several other new cases were detected.

The period following delivery was quite normal and without syncopal spells. The interrogation of the ICD still showed PVCs (6200 in 8 months) but there were fewer than on the ambulatory ECG recording during pregnancy. The amount of atrial pacing was <1%, but ventricular pacing was 2–4%, despite the programming of long AV‐delays. Over 7 years of follow‐up, no appropriate shocks for ventricular arrhythmias and no inappropriate shocks or other complications have occurred.

Four years after the first genetic evaluation, the patient was invited to participate in a research project for enhanced genetic analyzes. The outcome was astonishing – a second mutation associated with LQTS 1, KCNQ1‐mutation c.217C>A, p.P73T, was found. In addition to LQTS type 3, it was clear she was now coping with LQTS type 1, that is, she had the more severe disease involving double mutations. While she was protected from SCD and other potentially dangerous arrhythmias by her ICD, another evaluation of family members was warranted in order to determine their susceptibility to SCD. Although she tolerated the ICD, she found her overall situation to be very stressful. She obtained sick leave from her administrative work, despite support from family and healthcare professionals.

## Discussion

This case study reports on the complex management of LQTS with regard to differential diagnosis between cardiac syncope and epilepsy; it was further complicated by concomitant bradycardia, and risk stratification of SCD, ICD implant during pregnancy, technical considerations regarding the choice of ICD and programming options, pharmacological considerations, genetic complexity cascade screening, along with lifestyle modifications, and coping strategies.

**Figure 3:**
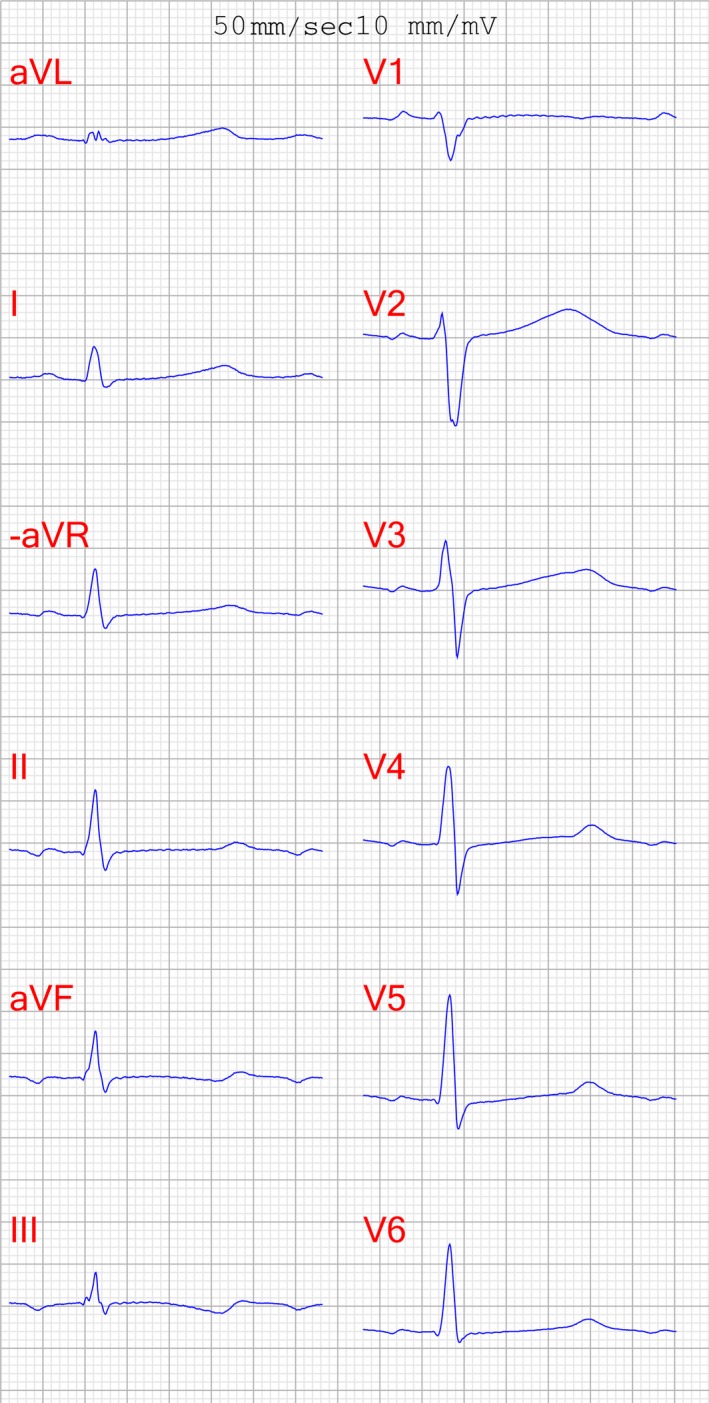
ECG with QTc 470 ms (without terbutaline but on phenytoin).

Long QT syndrome can be diagnosed when the QTc interval (corrected for heart rate) is ≥500 msec or when the patient has a risk score of ≥3.5 or when there is evidence of a disease‐causing mutation 3, 4, 5. Despite QT > 500 msec at ECG during childhood the diagnosis of LQTS had been overlooked in our patient. As an adult she also had an ECG with QT = 470 msec (Fig. 3), which shows the dynamic prolongation of the QT interval. If a patient with unexplained syncope has a QTc that falls between 480 and 499 msec in repeated 12‐lead ECGs, the diagnosis of LQTS can likewise be made 3, 4. The longest QT interval measured by any of the 12 leads should be used for diagnosis; typically, this will be the precordial leads V_2_ or V_3_. Should there be a >40 msec difference between two adjacent leads, then another adjacent lead should be used for measurement 6, 7. It is important to rule out alternative explanations for QT prolongation. Such causes might include acquired myocardial conduction delays or low levels of potassium, magnesium, or calcium, as well as taking QT‐prolonging drugs 5, 8. Patients should be interviewed about their actual drug regimens to determine if they are taking any drugs that might prolong the QT interval. Adrenergic response is known prolong the QT interval, and in this case terbutaline unmasked the LQTS and led to diagnosis 9.

The patient had been diagnosed with epilepsy as a child. There are several clinical diagnostic clues that can help to differentiate epilepsy from cardiac syncope. Epilepsy is characterized by certain symptoms, which differ from syncopal episodes: Epileptics are more likely to have prodromal symptoms (aura), longer duration of episodes, lateral tongue bite, deviation of the skull, involuntary micturition, and seizures with convulsions that leave the patient tired 10. However, there is a considerable overlap in symptomatology and life‐threatening arrhythmias may result in cerebral anoxia followed by convulsions, micturition, and fatigue 10. In a study on sudden unexpected death attributed to epilepsy, 7% of patients had a genotype of LQTS, and a total of 21% of had a mutation in a dominant cardiac arrhythmia gene 11. Moreover, there is a speculation that mutations in the sodium channels co‐expressed in both the heart and the brain may cause death 12. Interestingly, patients with both an epilepsy diagnosis combined with sodium channel mutations (e.g., Dravet syndrome) are known to have an increased risk of SCD 13 and to be susceptible to QT variability 14. However, our patient had a negative electroencephalogram, a history more typical for a cardiac disorder, and genetic mutations pathognomonic for LQTS.

Since the discovery of the molecular basis for LQTS in 1995, at least 15 associated genes have been identified, and about 90% of these mutations are found in KCNQ1, KCNH2, and SCN5A, causing types 1, 2, and 3, respectively, of LQTS 15. The three types of LQTS can be differentiated on an ECG. Type 1 is characterized by broad‐based T‐waves. Type 2 exhibits a low‐amplitude notched or biphasic T‐wave. Type 3 has a long isoelectric portion followed by a narrow‐based T‐wave. 16. LQTS is a relatively rare disorder with prevalence in live births of 1:2000 17. The decision to offer genetic testing in this individual case was based on ECG findings, unexplained symptoms, and a family history of SCD and was strongly supported by expert consensus (class I indication) 18. Once a molecular diagnosis of the patient is finalized, genetic counseling of biological relatives can be performed. This cascade screening is important to identify all cases in a family and offer individualized risk stratification, advice, and treatment. In this case, a few other relatives and the patient's son were found to have LQTS.

Lifestyle modifications for LQTS include avoiding strenuous exercise and other triggers, sometimes specific to the specific LQTS type. It is advisable to inform patients about situations where they risk experiencing electrolyte disturbances, that is, vomiting, diarrhea, and crash diets for weight loss 5. It is also crucial for patients, or parents of pediatric patients, as well as healthcare providers to be aware of drugs which may prolong the QT interval. We advocate the use of the webpage www.qtdrugs.org for updated information. Beta‐blockers are generally indicated in LQTS, even in genotypes with normal QT. According to guidelines there is no preference for a specific beta‐blocker as long it is long‐acting to avoid fluctuations in serum levels. It is important to counsel the patient to continue medication with any abrupt cessation; stopping the drug suddenly may trigger rebound mechanisms, which, in turn, may cause arrhythmias. However, there remain controversies regarding the beta‐blocker of choice 19. A beta‐blocker, preferably in a high but tolerable dosage, is advocated and seems to be an effective protection against SCD 5, 20, 21. Conversely, beta‐agonist treatment is associated with adverse outcome 22. In selected cases, left cardiac sympathetic denervation is recommended in high‐risk patients if beta‐blockers are not tolerated, are ineffective, or the patient refused an ICD 5. Other pharmacological therapies have been tried in case series with sometimes successful short‐term outcomes: mexiletine, flecainide, and ranolazine 23, 24, 25. Our patient was already taking phenytoin since she was 15 years old, and we decided to continue that based on phenytoin's pharmacological mechanisms, which could be favorable considering the molecular basis of her specific SCN5A mutation. After repeated consultations with a neurologist, the concomitant diagnosis of epilepsy could not be ruled out. Our case was further complicated by the presence of second‐degree AV block, described previously 26, 27. At ambulatory ECG monitoring, a second‐degree AV block was noticed and correlated with loss of stamina when climbing the stairs. This may be interpreted as functional AV block due to prolonged ventricular repolarization.

We decided to recommend a primary‐prevention ICD to this patient. Our patient had a very long QT, unexplained syncope in adulthood, and female sex, all of which have been shown to predict SCD in the International Long QT Syndrome Registry 28. Later, she was found to have dual mutations, which increase the risk for SCD, but this was not known when her ICD was recommended. Due to early reports of high mortality associated with LQTS type 3 and a comparatively high percentage of such patients in the European ICD Registry for ICD 29, the efficacy of beta‐blockade in LQTS type 3 has been under debate 19, 20. SCD at rest may have made clinicians more prone to prescribe ICD therapy, but current guidelines do not support this approach based on epidemiological studies on lifetime SCD event rates 30 where females with LQTS type 3 actually have less risk of SCD than their male counterparts. Pregnancy does not seem to be associated with higher risk for SCD, but the first 9 months postpartum may be a vulnerable period for the patient 31.

Family history of SCD, in and of itself, is not an indication for ICD therapy, but an ICD may be indicated based on the patient's phenotype as part of an overall risk assessment 5, 32. Thus, the death of our patient's mother at age 36 was not part of our formal risk stratification. Yet, her mother's untimely death convinced the patient that she was in danger of a potentially lethal outcome unless an ICD was implanted 5.

In 2009, when this case began, we decided to implant a transvenous ICD during pregnancy, requiring minimal ionization. Nowadays, a subcutaneous ICD would be the preferred alternative 33 if the patient does not require bradycardia pacing. In programming the ICD's parameter settings, it was important to take into account the often self‐terminating polymorphic ventricular tachycardias characteristic of LQTS, which would necessitate a longer detection interval. A long detection interval was programed in a single zone (high cutoff rate). This particular patient also exhibited severe anxiety, particularly in a hospital setting, so it was crucial to program the device to prevent inappropriate shocks. In addition to optimal device programming, a team of healthcare specialists cared for the patient. A holistic approach to ICD patients is important for their safety and well‐being 34.

## Conclusions

The management of LQTS may include several considerations with regard to differential diagnosis, dual mutations, risk stratification, ICD and may require long‐term holistic patient‐centered care.

## Conflict of Interest

None declared.

## Authorship

PM: involved in writing of the article. PEG: involved in revision of the article, management of patient. Both authors approved the final version of the case report for submission to the *Clinical Case Reports*.
